# Asciminib Use Highlighting Underlying Moyamoya Disease: A Case Report

**DOI:** 10.7759/cureus.63364

**Published:** 2024-06-28

**Authors:** Saloni Savani, Arpita Pawa, Naved Salim, Tithi Savani, Samip Master

**Affiliations:** 1 Internal Medicine, Willis-Knighton Health System, Shreveport, USA; 2 College of Medicine, Edward Via College of Osteopathic Medicine, Shreveport, USA; 3 Internal Medicine, Gujarat Medical Education and Research Society, Ahmedabad, IND; 4 Hematology and Oncology, Willis-Knighton Health System, Shreveport, USA

**Keywords:** stroke, tyrosine kinase receptor inhibitors, asciminib, chronic myeloid leukemia, moyamoya

## Abstract

We highlight here a case of Moyamoya disease (MMD) developed after treatment for chronic myeloid leukemia (CML). Moyamoya, a term meaning "a hazy puff of smoke" in Japanese, denotes a chronic occlusive cerebrovascular condition involving bilateral stenosis or closure of the terminal part of the internal carotid arteries (ICAs) and the proximal sections of the anterior cerebral arteries (ACAs) and middle cerebral arteries (MCAs) resulting in the development of abnormal vascular collaterals.

A 40-year-old African-American female with a past medical history of CML presented to the oncology clinic with expressive aphasia. Of note, she was diagnosed with CML eight years ago and was previously treated with dasatinib and nilotinib with only partial remission. She tested positive for the T315I mutation and was initiated on asciminib therapy about a month before her symptoms surfaced. Asciminib, an allosteric inhibitor targeting breakpoint cluster region-abelson murine leukemia 1 (BCR-ABL1) kinase activity, has gained approval for treating patients diagnosed with chronic-phase CML who have not responded to two prior lines of therapy or for those carrying the T315I mutation. During admission, the patient underwent brain magnetic resonance imaging (MRI) and a computed tomography (CT) angiogram of the head showed moderate to severe narrowing at the origins of the bilateral MCA and ACA, concerning Moyamoya syndrome. Although not classically associated with asciminib therapy, we report here a patient with CML who developed expressive aphasia one month after starting the medication. Due to the high index of suspicion, asciminib was discontinued, and the patient was referred for bone marrow transplant evaluation and concurrently started on cytarabine + peginterferon. The patient had improvement in her symptoms of aphasia after the drug was discontinued and returned to her baseline functional status.

No cardiovascular side effects associated with the use of asciminib are currently reported in the literature. However, we have described a case of such an occurrence. Therefore, extra caution should be taken in prescribing asciminib in patients with risk factors or a prior history of stroke.

## Introduction

Moyamoya disease (MMD) denotes a chronic occlusive cerebrovascular condition involving bilateral stenosis or closure of the terminal part of the internal carotid arteries (ICAs) and the proximal sections of the anterior cerebral arteries (ACAs) and middle cerebral arteries (MCAs), resulting in abnormal vascular collaterals. There are four presentations related to MMD: ischemic, epileptic, hemorrhagic, and other [1]. Moyamoya roughly translates to "a hazy puff of smoke" in Japanese. The collateral vessels formed to compensate for the progressive stenosis have a “hazy” or “smokey” appearance on cerebral angiographic imaging [2]. This disease is most prevalent in two different age ranges: approximately 10 years old and between 30 to 45 years old. Both children and adults commonly experience ischemic symptoms, including transient ischemic attacks (TIAs). However, adults are more prone to intracranial hemorrhages compared to children [3]. 

Asciminib is an allosteric inhibitor targeting breakpoint cluster region-abelson murine leukemia 1 (BCR-ABL1) kinase activity that has gained approval for treating patients diagnosed with chronic-phase chronic myeloid leukemia (CML) who have not responded to two prior lines of therapy or for those carrying the T315I mutation [4]. Common side effects of tyrosine kinase inhibitor (TKI) therapy include edema, nausea, hypothyroidism, vomiting, and diarrhea. TKI therapy has also been known to cause adverse cardiovascular effects such as hypertension, atrial fibrillation, and heart failure. Newer generation TKIs, such as nilotinib, have shown an increased association with vascular adverse events [5,6]. No literature currently reports microvascular or cerebrovascular events specifically associated with asciminib therapy.

This report aims to highlight the potential association between TKI therapy, specifically asciminib, and cardiovascular complications. We report here a patient with CML who developed expressive aphasia one month after being started on asciminib therapy and was found to have characteristic brain imaging findings consistent with MMD, introducing the possibility of the medication having a potential cardiovascular side effect profile.

## Case presentation

A 40-year-old African-American female with a past medical history of CML presented to the oncology clinic with expressive aphasia. She reported difficulty speaking but denied any other neurological symptoms like numbness, weakness, or tingling. These symptoms began a month prior and she was evaluated in the emergency room with a computed tomography (CT) scan of the head, which was negative for any acute abnormalities. During her clinic visit, she continued to experience word-finding difficulty, and the decision was made to admit the patient for a full neurological workup. The patient was diagnosed with CML eight years ago and was previously treated with dasatinib and nilotinib with only partial remission. Further genetic testing was performed, which indicated that she was positive for the T315I mutation (Table 1). Following this, the patient was initiated on a specifically targeting ABL myristoyl pocket (STAMP) inhibitor, asciminib, about a month before her symptoms began.

**Table 1:** Gene mutation analysis This patient has a previously documented p210 BCR-ABL1 transcript type. This assay detects acquired mutations in the kinase domain region of BCR-ABL1 oncogene (ABL1 amino acids 218-401; cDNA reference GRCh37(hg19)NM_005157.5), which have been associated with significant clinical or in vitro resistance to TKI therapy. The presence of KDM was evaluated by Sanger sequencing of the ABL1 region. If the BCR-ABL1 quantitative PCR level is very low, RT-PCR amplification of BCR-ABL1 mRNA may be unsuccessful in this assay. In general, a normalized BCR-ABL1 quantitative level above 0.1% (international scale) is required in order to detect a KDM by this assay [7-11]. KDM: kinase domain mutations; WB EDTA: whole blood ethylenediaminetetraacetic acid; BCR-ABL: breakpoint cluster region-abelson murine leukemia 1; VC: ascorbic acid

	Results
BCR-ABL Specimen Type, Blood	WB EDTA (VC)
BCR-ABL Fusion Form, Blood	210 (VC)
BCR-ABL Final Diagnosis, Blood	See below
Comment:	Peripheral blood, BCR-ABL1 kinase domain mutation analysis: positive. Mutation identified in the BCR-ABL 1 kinase domain region. This mutation and its corresponding amino acid change is g. 133748283CT; p.Thr315lle (T315I). The T315I mutation is associated with pan-resistance to nearly all TKIs but is sensitive to ponatinib and asciminib

**Figure 1:**
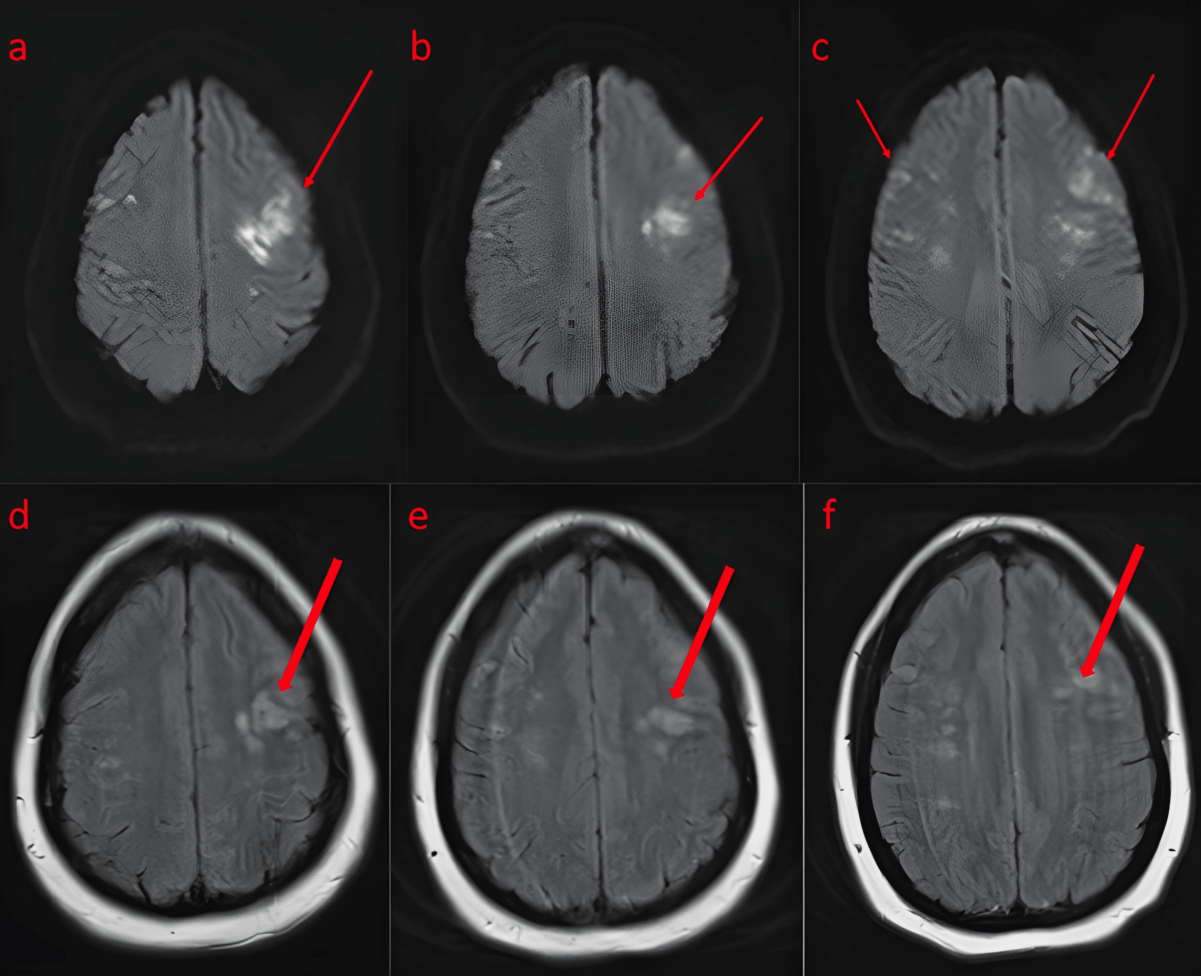
MRI brain with diffusion-weighted imaging sequence showing multiple foci of cortical/subcortical restricted diffusion in the cerebral hemispheres bilaterally, including the temporal lobes (a) and subcortical regions (b). Lesions were found predominantly in the frontal lobes (c) within the watershed distribution, compatible with acute/subacute infarctions. Also shown are FLAIR images (d,e,f), which display acute/subacute infarcts MRI: magnetic resonance imaging

During her admission, the patient underwent a brain magnetic resonance imaging (MRI) that revealed multiple foci of cortical/subcortical restricted diffusion in the cerebral hemispheres bilaterally, predominantly in the frontal lobes, with findings compatible with acute/subacute infarction (Figure 1).

**Figure 2:**
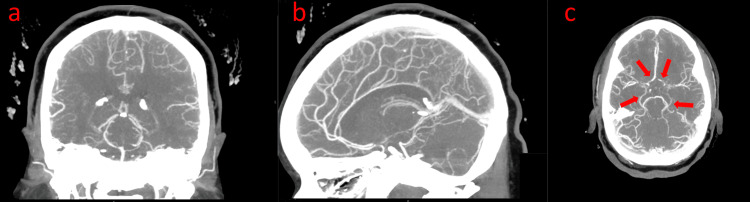
CT angiogram head and neck: moderate to severe narrowing at the origins of the bilateral MCA M1 segment and ACA A2 segment, which were patent distally. (a) Coronal view, (b) sagittal view, and (c) transverse view (red arrows identifying affected areas) CT: computed tomography; MCA: middle cerebral artery; ACA: anterior cerebral artery

A CT angiogram of the head showed moderate to severe narrowing at the origins of the bilateral MCA-M1 segment and ACA-A2 segment, which were patent distally, concerning Moyamoya syndrome (Figure 2).

Given the lack of cardiovascular risk factors, underlying vasculitis was considered a differential, and a battery of autoimmune investigations was sent, all of which were negative. Additionally, these imaging findings were consistent with that of MMD. The patient followed closely with neurology and oncology. Due to the high index of suspicion, asciminib was discontinued, and the patient was referred for bone marrow transplant evaluation and concurrently started on cytarabine + peginterferon. The patient had improvement in her symptoms of aphasia after the drug was discontinued and returned to her baseline functional status. 

## Discussion

Asciminib, a STAMP inhibitor, has been used in patients with chronic-phase CML resistance or the setting of intolerance to ≥2 prior TKIs and those with T315I mutation [12]. The emergence of the T315I mutation stands as the primary resistance mechanism observed in patients with CML against both first- and second-generation TKIs [13]. T315I mutation is a change in the BCR-ABL fusion protein that confers resistance to most TKIs necessitating the use of other agents. Asciminib attaches to a myristoyl site on the BCR-ABL1 protein. This binding action effectively traps BCR-ABL1 in an inactive state, employing a mechanism different from other ABL kinase inhibitors [11]. Over the past decade, ponatinib has been the primary treatment choice in this scenario. However, when administered at its maximum dose (45 mg/day), there is a considerable risk of serious vascular occlusive events. Lower doses reduce this risk but also diminish effectiveness. These recent therapeutic advancements mean that for many resistant chronic-phase CML patients who have failed multiple TKIs, two options are now available: adjusted-dose ponatinib and asciminib [14]. 

Asciminib treatment continues until either the disease worsens or side effects become intolerable. Side effects are more common with higher doses and affect almost all patients. These side effects include anemia, neutropenia, muscle pain, fatigue, headache, rash, and diarrhea. Some severe but less common adverse events may occur such as severe bone marrow suppression, pancreatitis, high blood pressure, hypersensitivity reactions, embryo-fetal toxicity, and cardiovascular toxicity [15].

MMD is a chronic condition characterized by bilateral progressive narrowing of the terminal intracranial portion of the ICA and the circle of Willis. Its underlying cause remains unknown. MMD is often associated with inherited conditions such as sickle cell disease or trait, down syndrome, and neurofibromatosis type 1 as well as acquired conditions including head and/or neck irradiation, chronic meningitis, skull base tumors, atherosclerosis of skull base arteries, arteriosclerosis, and cerebral vasculitis. MMD exhibits a bimodal age distribution with the first peak occurring during the first decade of life and the second peak occurring in the fourth decade. The most frequent manifestation in Moyamoya patients is cerebral ischemic events such as TIAs or ischemic infarcts. Intracerebral hemorrhage predominantly occurs in adult patients with MMD. Seizures can occur in both adults and children. Symptoms can be classified based on their cause: those resulting from cerebral ischemia (such as stroke, TIA, and seizures) and those arising from the development of collateral vessels that compensate for ischemia (such as hemorrhage and headaches) [16].

New generation TKIs, including nilotinib and ponatinib, have been shown to cause cerebrovascular complications, including ischemic stroke [17]. Our report here represents a case of underlying MMD, which was unmasked in a patient recently initiated on asciminib therapy. Whether the patient had developed MMD secondary to underlying stroke risk factors or the previous use of TKIs known to predispose to stroke remains uncertain. The literature on the association between this medication and cardiovascular side effects is sparse [18]. However, our report highlights the potential for the drug to cause significant cardiovascular side effects.

Given our findings, patients placed on TKI therapy for CML need to be managed with extra precautions. This is especially important in cases involving patients with other comorbidities for stroke including renal disease, diabetes, hypertension, and cancer. Lifestyle modification should be emphasized in these patients. Optimal blood pressure control may also be necessary to mitigate the risk of developing MMD in patients started on TKI therapy.

## Conclusions

This case underscores the importance of exercising extra caution when considering asciminib treatment for patients with pre-existing stroke risk factors or a history of stroke. As such, careful observation and expectant management are imperative to ensure patient safety and optimize therapeutic outcomes.
